# A Repeatability Study of Measurement of Micturition With Voiding Sonography and Uroflowmetry of Asymptomatic Women

**DOI:** 10.1111/luts.70020

**Published:** 2025-07-08

**Authors:** Bernadette Dellar, Ryan Stafford, Eric Chung, Gabriel Schaer, Margret Sherburn, Roxanna Turner, Handoo Rhee, Anna Page, Paul W. Hodges

**Affiliations:** ^1^ School of Health and Rehabilitation Sciences The University of Queensland Brisbane Australia; ^2^ Faculty of Medicine The University of Queensland Brisbane Australia; ^3^ Department of Gynaecology The University Hospital of Zurich Zürich Switzerland; ^4^ Department of Physiotherapy The University of Melbourne Melbourne Australia; ^5^ Queensland Radiology Specialists Brisbane Australia; ^6^ Evelina Children's Hospital Guys and St Thomas' NHS Foundation UK

**Keywords:** diagnostic techniques, gynecology, lower urinary tract symptoms, pelvic floor disorders, ultrasound, urethra, urination, urology

## Abstract

**Aims:**

This study aimed to assess the intra‐ and inter‐tester repeatability of voiding sonography measures in asymptomatic women and to examine the effect of perineal transducer placement on uroflowmetry.

**Methods:**

A prospective observational study of 32 asymptomatic women was conducted using ultrasound with simultaneous uroflowmetry. Participants completed four voids (two per day). The ultrasound transducer placed on the perineum assessed bladder neck and urethral displacement during micturition. On Day 1, a void with ultrasound and uroflow recorded by a sonographer was compared to a void recorded with uroflowmetry only to determine whether the transducer placement affected voiding. On Day 2, two sonographers evaluated the voids with ultrasound and uroflowmetry. Each sonographer measured their own images. One sonographer also made measures on the images recorded by the second sonographer. Uroflowmetry measures and void patterns were evaluated. The repeatability of ultrasound measures was analyzed using intraclass correlation coefficient (ICC 2,1) and Bland–Altman analysis, and *t* tests examined the transducer's impact on uroflowmetry.

**Results:**

Some changes in flow rate and flow pattern (20% of participants) were observed with placement of the transducer. Most sonographic measures showed good to excellent inter‐ and intra‐tester repeatability, and between measures made by the different testers on the same image. During void, bladder neck and urethral diameter were more repeatable if measures were made at the time of maximum flow estimated from the simultaneous uroflowmetry.

**Conclusion:**

Voiding sonography is both feasible and repeatable. Perineal transducer placement affected micturition for some individuals. Voiding sonography combined with uroflowmetry shows promise for a noninvasive urogynecology functional assessment.

## 1 Introduction

Voiding dysfunction (VD) and dysfunctional voiding (DV) are conditions that disrupt normal bladder emptying. VD is diagnosed through symptoms and urodynamics and is defined by slow and/or incomplete micturition [1]. VD is a common, underdiagnosed with a reported incidence of 6%–23% [2, 3]. Mechanisms of VD include changes in anatomical, functional, and neurological aspects of micturition, dysfunction of pelvic floor muscle coordination [4], detrusor underactivity/acontractility, and/or bladder outlet obstruction [3]. Anatomical causes of obstruction can be extrinsic (e.g., constipation, cancer, fibroids, pregnancy, prolapse), intrinsic (e.g., urethral strictures, diverticulum, foreign bodies), and iatrogenic (e.g., surgical repairs, trauma, prolonged catheterization) [5, 6]. DV is defined as “intermittent and/or fluctuating flow rate due to involuntary intermittent contractions of the periurethral striated muscle during voiding in neurologically normal individuals”. If there is a neurological cause of DV, this is referred to as detrusor sphincter dyssynergia [1]. Although physical examination is important, there are no global consensus‐based diagnostic criteria for female VD and DV, and patient symptoms alone can be unreliable [7].

Effective management of DV and VD requires assessment of underlying mechanisms and pathophysiology. Current assessment methods are either invasive (e.g., catheter insertion) [8] or limited (e.g., noninvasive uroflowmetry which shows flow data but not dysfunction mechanisms). Uroflowmetry is a screening tool but is limited by variable definitions [9] and requires additional testing [4]. Pressure flow studies offer valuable information, but bladder and urethra catheters affect micturition patterns in some patients [8]. Combined noninvasive methods may provide clearer insight into mechanisms.

Voiding sonography (VS) is a new ultrasound method to assess voiding [10]. In combination with uroflowmetry, it could offer valuable clinical insights. Before VS can be implemented, it is important to assess whether it affects voiding and if the measures are repeatable.

This study aimed to investigate: (i) the impact of the VS procedure on voiding (measured with uroflowmetry), (ii) intra‐tester repeatability of VS with the same assessor replicating the procedure on separate days, (iii) inter‐tester repeatability with two assessors replicating the procedure on the same day (with bladder refilling), and (iv) inter‐tester repeatability of measures made on the same images.

## 2 Methods

### 2.1 Participants

Thirty‐five women were recruited via social media. Participants were excluded if they had: scores on the ICIQ‐UI SF and ICIQ‐VS that were greater than 12 and 6, respectively; a history of pelvic floor issues (including voiding or incontinence surgery); known neurological diagnosis; diabetes; or unable to void in each of the four sessions. Three participants were unable to void during the procedure and were excluded. Participants provided written and informed consent.

### 2.2 Sonographers

Ultrasound imaging was performed by two sonographers. Sonographer A(BD) had 15 years of experience including development of this technique. Sonographer B(RT) had 5 years of experience in general sonography, but no prior experience in this technique. For training, RT observed BD perform two scans. BD supervised RT to perform the first five scans. Some minor corrections were made to the placement of the transducer and image criteria at these sessions. Data from these sessions are included in the results.

### 2.3 Ultrasound Imaging

A General Electric (GE) ultrasound machine (E8 model) with a C1‐5, 1–5 MHz frequency, 2D convex footprint with a 70° wide field of view transducer was used. The US machine in‐built measurement requirements include distances in mm (to one decimal place) and measurement of angles via a caliper tool; a Cartesian *X* and *Y* axis display references, and the capacity to record more than 90s of continuous DICOM video.

### 2.4 Uroflowmetry Device

A portable wireless UROCAP IV uroflowmetry system, with Bluetooth technology and integrated with the Goby UDS software, was used to record urine flow. Data were extracted for maximum flow rate, average flow, void volume, and duration of void using the manufacturer's software. Flow patterns were recorded and exported for categorization.

### 2.5 Participant Preparation

The initial contact with the participant was via a phone call to explain the procedure in detail. Participants were aware that the sonographer would be in the room and holding the transducer against their perineum during voiding.

### 2.6 Procedure

Participants followed a specified bladder filling [11] protocol that started 3.5 h before the appointment, drinking 250 mL of fluid (water, juice, or non‐diuretic causing liquid) at hourly intervals (total—750 mL) and arrived with a self‐reported full bladder. Bladder fullness was established with a standard transabdominal ultrasound method, in a supine position when the participant indicated the urge to void. The volume of the bladder was calculated with the standard three measures in two planes [12].

**FIGURE 1:**
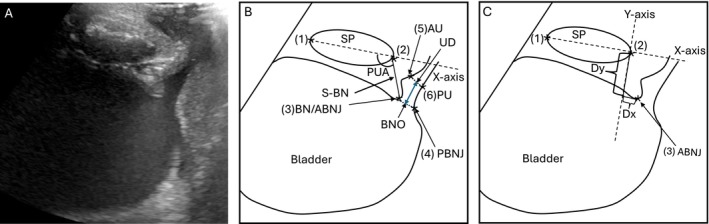
(A) Ultrasound image at peak (max) flow with bladder neck and urethra opened. (B) Line drawing from (A) showing recommended measures. Symphysis pubis (SP) length—distance between “*” at (1) to (2) is used to confirm the reproducibility of image plane; symphysis to bladder neck (BN) distance (S‐BN)—the distance from the inferior margin of the fibrocartilage SP (2) to the anterior bladder neck junction (ABNJ) (3); pubourethral angle (PUA)—angle between the dotted line bisecting the SP at (1) to (2) and the S‐BN line at (2) to (3); urethral diameter UD—measured at 10 mm from BNO (blue line) as the perpendicular distance between inner surface of the anterior wall (AU) and inner surface of the posterior wall of the urethral opening (PU), and the BN diameter or opening (BNO)—distance between ABNJ at (3) and posterior bladder neck junction (PBNJ) at (4). (C) Line drawing from (A) showing additional recommended measures. Dx—*x*‐axis Cartesian co‐ordinate of the ABNJ location relative to SP at (2); Dy—*y*‐axis Cartesian co‐ordinate of the ABNJ location relative to SP at (2). Adapted from Schaer et al. [13].

For the VS measurement, participants sat on a commode with the sonographer holding the covered (aseptic technique) transducer in a mid‐sagittal plane on the mons pubis (anterior boundary at the pubis boarder of the perineum). The soundwaves pass through the fibrocartilaginous symphysis pubis (SP) which served as a fixed landmark for reference against motion of other pelvic structures, as shown in Figure 1. As the participant voids, the bladder neck and urethra widen, and at the end of the void, the emptied bladder can be visualized. Post void residual (PVR) bladder volume was measured (transabdominally as described above) and quantified.

Participants attended two sessions and completed two voids in each session. Session 1 was conducted by BD. During this session, Void 1 consisted of simultaneous uroflowmetry and ultrasound recording. Following the bladder refilling procedure, which included drinking 300 mL of water and waiting either up to 3 h or when the participant had the urge to void. Void 2, flow parameters were recorded with uroflowmetry only (no ultrasound imaging). During Session 2, Void 3 was measured by BD, and after the participant refilled their bladder, Void 4 was measured by RT. Session two consisted of simultaneous ultrasound and uroflowmetry.

### 2.7 Uroflowmetry Analysis

Uroflowmetry data were analyzed in two ways. First, flow patterns were assessed. Five types of flow patterns were identified as described by Tarcan et al. [14]. The flow pattern does not guarantee diagnostic abnormality but is a guide to the existence of an underlying specific condition [15]. Three experienced assessors, BD (sonographer), HR (adult urologist), and AP (pediatric urologist), classified the flow patterns into five shapes: (1) bell; (2) tower; (3) fluctuating; (4) intermittent; and (5) plateau. No other criteria or instructions were given to the assessors. Second, the quantitative measures of (i) maximum flow, (ii) average flow, (iii) voided volume, and (iv) flow time were extracted.

### 2.8 Ultrasound Analysis

US videos were analyzed post‐procedure and images for measurements were selected by the sonographer who recorded the void. All measurements were performed and stored on the US machine. The following measures were made when the bladder was at full capacity, during voiding (at the time of widest bladder neck and urethral diameter); and at the end of micturition [10]

- SP length—distance between the superior and inferior margin along the mid‐pubic line (MPL).
- Pubourethral angle (PUA)—angle between the MPL to the anterior bladder neck junction (ABNJ). The ABNJ was selected because it was consistently visible [16]
- Symphysis to anterior bladder neck distance (S‐BN)—distance between the inferior margin of the SP to the ABNJ.
- X‐cartesian plane of the bladder neck (Dx)—the *x*‐axis distance between the ABNJ and the inferior border of the SP.
- Y‐cartesian plane of the bladder neck (Dy)—the *y*‐axis distance between the ABNJ and the inferior boarder of the SP.

Additional measures were made at the time of the widest bladder neck and urethral diameter (identified from visual inspection of the ultrasound video). Based on the results, measures were also made at the time of max (peak) flow (estimated from the uroflowmetry data). These measures were:

vi Bladder neck diameter (BND)—measured between the inner‐to‐inner walls of the ABNJ and PBNJ following the curvature of the bladder.
vii Urethral diameter (UD)—measured perpendicular to the course of the urethra and between the inner‐to‐inner walls.

### 2.9 Statistical Analysis

Descriptive statistics of participant demographics, paired *t* test, and Bland–Altman plots were calculated in Excel; two‐way, mixed effects model; absolute agreement; single measure; intraclass correlation coefficients (ICC) calculations were performed using SPSS (version 29; IBM).

To determine whether the application of the ultrasound transducer to the perineum affected flow, we compared the flow pattern and quantitative flow measures from the uroflow between voids with (Void 1) and without (Void 2) ultrasound. Allocation of pattern between assessors and between different voids (e.g., with and without the transducer on the perineum) was considered as “agreed” if the same pattern was identified by all the assessors; “minor” difference if the pattern changed from bell to tower, fluctuating to intermittent, or if the difference in pattern was only identified by one assessor; and “major” difference if the pattern changed from bell/tower to fluctuating/intermittent/plateau and the difference was categorized by two assessors. Quantitative data were compared between voids using paired sample *t* tests.

Intra‐ and inter‐tester repeatability of ultrasound measures was assessed with two‐way mixed, absolute agreement, ICC (1,2) and Bland–Altman analysis. ICCs were calculated to compare: (i) measurements made by one sonographer on the same participant on different days (Void 1 vs. 3); (ii) measurements made by two sonographers on the same participant on the same day (after bladder refilling; Void 3 vs. 4); and (iii) measurements made by two sonographers from the same video recording (Void 4). The ICC value is between 0 and 1 and repeatability was interpreted as: poor (< 0.20), fair (> 0.20–0.40), moderate (> 0.40–0.60), good (> 0.60–0.80), and very good or excellent (> 0.80) [17].

Bland–Altman analysis determined the limits of absolute agreement between the same measurement parameter for different voids. Bland–Altman analyses were based on mean difference and error estimates at the 95% confidence levels and any systematic bias between the two sonographers' ultrasound measures.

## 3 Results

### 3.1 Participants

Thirty‐five asymptomatic women volunteered. Two participants could not void with the ultrasound transducer on the perineum, and the third participant did not complete the first session due to an unrelated medical issue. The remaining 32 participants completed all four voids on 2 days. Characteristics of the 32 participants are presented in Table 1. Of the 126 voids performed, 88 (70%) involved complete bladder emptying and 38 (30%) voids had PVR of < 50 mL. Spillage of urine for two participants meant that uroflow data are only available for 30 participants for comparisons of measures with and without the ultrasound transducer. We were able to visualize the relevant structures and make the measures with VS in all participants.

**TABLE 1:** Demographic, uroflow, and ultrasound measures.

Parameter		Median	Range
Age (years)		34	(18–53)
BMI (kg/m^2^)		22.4	(18–35)
Parity		19 nulliparous 13 (with 1 or more child)	
ICIQ‐UI‐SF (count)		3	(0–10)
ICIQ‐VS vaginal symptoms (count)		1	(0–6)
ICIQ‐VS bothersome score (count)		0	(0–17)

Uroflow	Mean/median (SD)	Min–max (range)
Max flow rate (mL/s)	32/30 (11)	12–73 (61)
Average flow rate (mL/s)	15/15 (6)	6–34 (28)
Total output volume (mL)	461/448 (121)	214–1030 (816)
Flow time (s)	35/31 (16)	9–91 (82)

Ultrasound (*n* = 32)		Mean/median (SD)	Min–max (range)
SP (mm)	At full bladder	38.2/37.6 (4.1)	30.6–46.8 (16.2)
	Max void	37.9/37.6 (4.1)	31.7–45.1 (13.4)
	End flow	38.1/38.3 (3.8)	31.1–45 (13.9)
PUA (degrees)	At full bladder	120.3/118.7 (17.8)	79.3–154.7 (75.4)
	Max void	121.4/121.2 (17.9)	68.1–156.5 (88.4)
	End flow	125.3/125.1 (18.4)	81.2–155.3 (74.1)
S‐BN (mm)	At full bladder	26/26 (3.3)	16.6–32.1 (15.5)
	Max void	24.7/25.2 (4.2)	9.9–31.6 (21.7)
	End flow	27.1/27.4 (4)	17.4–34.7 (17.3)
Dx (mm)	At full bladder	13.5/14.1 (6.8)	−4.5 – 27.8 (23.3)
	Max void	12.7/13.7 (6.7)	−8.6 – 24.7 (16.1)
	End flow	14.6/16.4 (6.9)	−5 – 26.3 (21.3)
Dy (mm)	At full bladder	20.8/21.8 (5.2)	9.9–27.8 (17.9)
	Max void	19.7/21.1 (5.7)	3.9–27.6 (23.7)
	End flow	21.1/29.9 (6.4)	11.2–34.4 (23.2)
BND (mm)	Uroflow max flow	7.4/7.2 (3.6)	1.4–17 (15.6)
UD (mm)	Uroflow max flow	4.9/4.7 (1.6)	2.2–8 (5.8)

### 3.2 Uroflowmetry

#### 3.2.1 Evaluation of the Impact of Transducer Placement on Voiding—Flow Pattern

Classification of the participants' void pattern showed that independent assessors agreed on the pattern for 11/30 (37%) of Void 1, reported a minor difference for 15/30 (50%); and a major difference for 4/30 (13%). These differences were explained by variation in subjective interpretation of specific features of flow pattern (e.g., amplitude of peak which is used to distinguish between “bell” vs. “tower”). Comparison of pattern between voids with (Void 1) and without the transducer (Void 2) (each assessor's assessment compared to the pattern they identified in Void 1) showed agreement for 13/30 (43%), minor difference for 11/30 (37%), and major difference for 6/30 (20%). Detailed classifications of void patterns with and without the transducer on the perineum are presented in Data S1 and S2. The 20% of voids with major difference included change from “bell” to “plateau/fluctuating/intermittent.”

#### 3.2.2 Evaluation of the Impact of Transducer Placement on Voiding—Flow Parameters

Comparison of the quantitative measures between Void 1 and 2 with paired‐sample *t* tests showed that voiding with the ultrasound transducer had a slower average (*p* = 0.005; medium effect *d* = −0.55) and max flow (*p* = 0.003; medium effect *d* = 0.6); longer time (*p* = 0.002; medium effect *d* = −0.61), but no difference in void volume (*p* = 0.17; small effect *d* = −0.26) (Table 2). Taking together, the qualitative and quantitative data indicates placement of the transducer impacts voiding for approximately 20% of participants.

**TABLE 2:** Paired *t* tests of uroflowmetry parameters between with (Void 1 and Void 4) and without ultrasound imaging (Void 2).

Uroflowmetry measures	Void 2		Void 1		*t*(29)	*p*	Cohen's *d*	95% CI	
	*M*	SD	*M*	SD				Lower	Upper
*Q*_max_ (mL/s)	36.6	12.6	31.6	9.5	3.3	0.003	0.60	0.21	0.99
Average flow (mL/s)	18.6	6.7	15.8	6.0	3.0	0.005	0.55	0.17	0.93
Void volume (mL)	428.8	84.3	463.1	112.3	−1.4	0.167	−0.26	−0.62	0.11
Total void time (s)	26.6	13.5	33.6	13.0	−3.3	0.002	−0.61	−0.99	−0.21

Uroflowmetry measures	Void 2		Void 4		*t*(30)	*p*	Cohen's *d*	95% CI	
	*M*	SD	*M*	SD				Lower	Upper
*Q*_max_ (mL/s)	36.6	12.6	30.3	11.0	4.3	< 0.001	0.77	0.36	1.17
Average flow (mL/s)	18.6	6.7	13.7	5.5	7.0	< 0.001	1.25	0.77	1.72
Void volume (mL)	428.8	84.3	480.1	157.5	−1.9	0.072	−0.34	−0.70	0.03
Total void time (s)	26.6	13.5	39.3	17.9	−4.8	< 0.001	−0.87	−1.28	−0.45

*Note:* Void 1 (performed on Day 1 by BD), Void 3 (performed on Day 2 by BD), and Void 4 (performed on Day 2 by RT) poor—< 0.20, fair—0.21–0.40, moderate—> 0.40–0.60, good—0.60–0.80, and very good or excellent—0.81–1.0.

Comparison of void with and without the transducer for the second sonographer (note: the void without the transducer was performed on Day 1) showed similar differences between max flow (*p* < 0.001, medium effect *d* = 0.77), average flow (*p* < 0.001, large effect *d* = 1.25) and flow time (*p* < 0.001, large effect *d* = −0.87) and no difference in void volume (*p* = 0.072, small effect *d* = −0.34) (Table 2).

### 3.3 Voiding Sonography

#### 3.3.1 Intra‐Tester Repeatability Between Days

Data S4 presents the ICC data for intra‐tester repeatability. At full bladder, PUA and S‐BN measures demonstrated good repeatability (ICC 0.68); whereas SP, Dx, and Dy were moderate (ICC 0.57–0.63). At end void, PUA, Dx, and Dy had good (ICC 0.77–0.82) repeatability, S‐BN was moderate (ICC 0.60), and SP was fair (ICC 0.35). At the time of widest urethral diameter, PUA, S‐BN, Dx, and Dy data demonstrated good (ICC 0.61–0.80) repeatability, SP was moderate (ICC: 0.53), and BND and UD were fair (ICC 0.34 and 0.39, respectively). BND and UD measurements were reassessed at the time of maximum flow rate estimated from the uroflowmetry data. At this timepoint, the intra‐tester repeatability for BND and UD was fair to moderate (ICC 0.38 and 0.54, respectively). Data S3 presents the results from Bland–Altman limits of agreement at 95% CI and the calculated biases. The only appreciable bias was for PUA at full bladder which demonstrated a bias of 2.3 (Void 1 higher).

#### 3.3.2 Inter‐Tester Repeatability on the Same Day

Table 3 presents the ICC data for inter‐tester repeatability. At full bladder, the PUA, S‐BN, Dx, and Dy measurements demonstrated good repeatability (ICC 0.73–0.77), whereas the repeatability of SP was fair (ICC 0.22). At end void, PUA, S–BN, Dx, and Dy demonstrated good repeatability (ICC 0.65–0.68), and SP was poor (ICC 0.19). At widest urethral diameter, PUA and Dy measurements demonstrated excellent repeatability (ICC 0.85 and 0.81), Dx and S‐BN were good (ICC 0.80 and 0.66), whereas SP was fair (ICC 0.31). BND and UD repeatability were fair (ICC 0.24 and 0.37). When the BND and UD measures were repeated at max void identified from the uroflow, the repeatability of BND (ICC 0.39) and UD (ICC 0.37) improved slightly. Data S3 presents results for Bland–Altman analysis. Data only demonstrate appreciable bias for PUA at full bladder (−5.3) and at end void (−2.6).

**TABLE 3:** Inter tester repeatability data and ICC values of sonographic measures of micturition on the same day (*n* = 32) between Void 3 and Void 4 (two rater).

	Parameter	Void 3 mean (range) median (SD)	Void 4 mean (range) median (SD)	ICC
At full bladder	SP (mm)	38.7 (30–46.3) 38.3 (3.4)	40.1 (35.1–45) 39.9 (2.6)	0.20
	PUA (degrees)	118 (87.4–163.5) 117.4 (17.5)	123.3 (79.6–160.6) 123.4 (17.6)	0.72
	S‐BN (mm)	26.5 (21.5–36) 26.1 (3.7)	26 (19.1–33.5) 25.9 (3.6)	0.78
	Dx (mm)	12.2 (−1.2–30.2) 11.6 (7)	13.3 (−5.1–28.7) 13.5 (7)	0.74
	Dy (mm)	22.1 (8.7–30.9) 22.7 (5.1)	20.9 (10.5–28.4) 21.8 (4.8)	0.76
At widest urethral diameter	SP (mm)	38.3 (32–45) 37.8 (3.2)	39.5 (32.6–44.6) 39.8 (2.7)	0.29
	PUA (degrees)	121.9 (69.7–172.4) 120.9 (19.4)	121.9 (69.4–163.1) 121.1 (19.3)	0.86
	S‐BN (mm)	24.5 (18.2–31.6) 24.1 (3.6)	23.8 (12.1–31) 23.6 (4)	0.66
	Dx (mm)	12.5 (−8.3–29.5) 13.3 (6.9)	12.5 (−8.2–29.5) 12.4 (7.5)	0.81
	Dy (mm)	19.5 (4–28.4) 20.8 (5.4)	18.9 (9.3–26.7) 19.6 (4.6)	0.81
At max flow	BND (mm)	7.8 (2.8–13.3) 7.5 (2.8)	8.8 (4.2–15.1) 8.5 (2.1)	0.39
	UD (mm)	5.1 (1.0–9.3) 5.2 (2.3)	5.4 (4.2–9.1) 5.1 (1.5)	0.37
At end void	SP (mm)	39.8 (32–46.2) 40.4 (3.9)	39.3 (31.8–45.7) 39.2 (2.8)	0.19
	PUA (degrees)	125.1 (81.8–156.5) 126.3 (17.3)	127.7 (85.3–155.7) 128.6 (18.4)	0.65
	S‐BN (mm)	26.3 (19.2–33) 26.3 (3.3)	26 (18.6–33.9) 25.9 (4.1)	0.69
	Dx (mm)	14.7 (−3.2 to 29.4) 15.7 (7)	15.2 (−1.5–27.7) 16.2 (6.6)	0.68
	Dy (mm)	20.8 (10.9–32) 21.2 (5.2)	19.5 (9.6–32) 19.3 (6.1)	0.64

*Note:* Void 1 (performed on Day 1 by BD), Void 3 (performed on Day 2 by BD), and Void 4 (performed on Day 2 by RT) poor—< 0.20, fair—0.21–0.40, moderate—> 0.40–0.60, good—0.60–0.80, and very good or excellent—0.81–1.00.

#### 3.3.3 Inter‐Tester Repeatability of Sonographic Measures by the Two Assessors on Same Video

Data S5 presents the ICC data for inter‐tester repeatability of measures made from the same video/images. At full bladder, max flow, and end void, the PUA, S‐BN, Dx, and Dy measurements demonstrated good to excellent (ICC 0.76–0.91) repeatability, whereas SP was moderate to good (ICC 0.42–0.68), and BND and UD were good and moderate (ICC 0.66 and 0.51). These results indicate that most measures were reliably repeatable when the same images are measured by different sonographers. Again, repeatability improved when the BND (ICC 0.70) and UD (ICC 0.63) were measured at maximum flow. Data S3 presents results for Bland–Altman analysis, which demonstrate appreciable bias for PUA at full bladder (−3.6) and at end void (−2.5).

## 4 Discussion

The study demonstrates that noninvasive micturition assessment with VS is feasible and provides repeatable measures. Most participants voided similarly with and without the transducer, although some systematic differences in voiding rate were observed. VS, combined with uroflowmetry, holds promise for evaluating micturition. VS, with the transducer placed in contact with the perineum toward the anterior perineum, provides repeatable voiding measures. Combined with uroflowmetry, VS shows promise for noninvasive lower urinary tract assessment. Further research in symptomatic patients is needed to assess its clinical use, particularly when video fluoroscopy is unavailable or urodynamics fail to provide a diagnosis.

### 4.1 Impact of Transducer Placement

If VS alters micturition patterns, it could impact the assessment's validity. We found that voiding was systematically slower with the ultrasound transducer, supporting fundings from urodynamics that report slower voiding (longer duration, lower maximum flow rate) compared to uroflowmetry [1]. We observed some variation in flow patterns. Variation in flow patterns [7] has been linked to clinician experience and interpretation [18, 19] and clinical symptoms [20]. The observed changes in flow pattern in six women were minor, such as shifts from “bell” to “plateau,” “fluctuating” or “interrupted,” and one improved from “fluctuating” to “bell.” Although consistent flow pattern definitions could improve reliability [9], studies on interobserver agreement in uroflowmetry shown poor to fair results [18], despite clear definitions [15, 19]. Variations like a slight fluctuation at the end of a “bell” pattern could led to different classifications (e.g., “interrupted”). Additionally, pressure from the ultrasound transducer may explain some changes in voiding patterns, and only one assessor knew participants were asymptomatic.

### 4.2 Comparison With Previous Data

Our uroflowmetry measures aligned with findings from a systematic review and meta‐analysis [21]. Our average and maximum flow rates and flow time (15 mL/s, 32 mL/s, 35 s, respectively) are within the reported ranges (9–24 mL/s, 20–49 mL/s, 24–38 s). Our output volume of 461 mL exceeds the reported range of 224–409 mL, likely due to our bladder filling protocol.

Although no participants reported voiding symptoms, not all showed a traditional “bell” pattern which is considered “normal.” Other patterns have been reported in asymptomatic participants. A study comparing uroflowmetry and video‐urodynamics in 10 asymptomatic women found repeatable flow patterns in seven, with only four showing a “bell” pattern [20].

No prior study used the same transducer placement, so direct comparison with ultrasound outcomes is not possible. However, one study using a transvaginal transducer reported a similar S‐BN distance to our findings (27.1m vs. 25.7 mm) [22].

### 4.3 Repeatability of VS Measures

**FIGURE 2:**
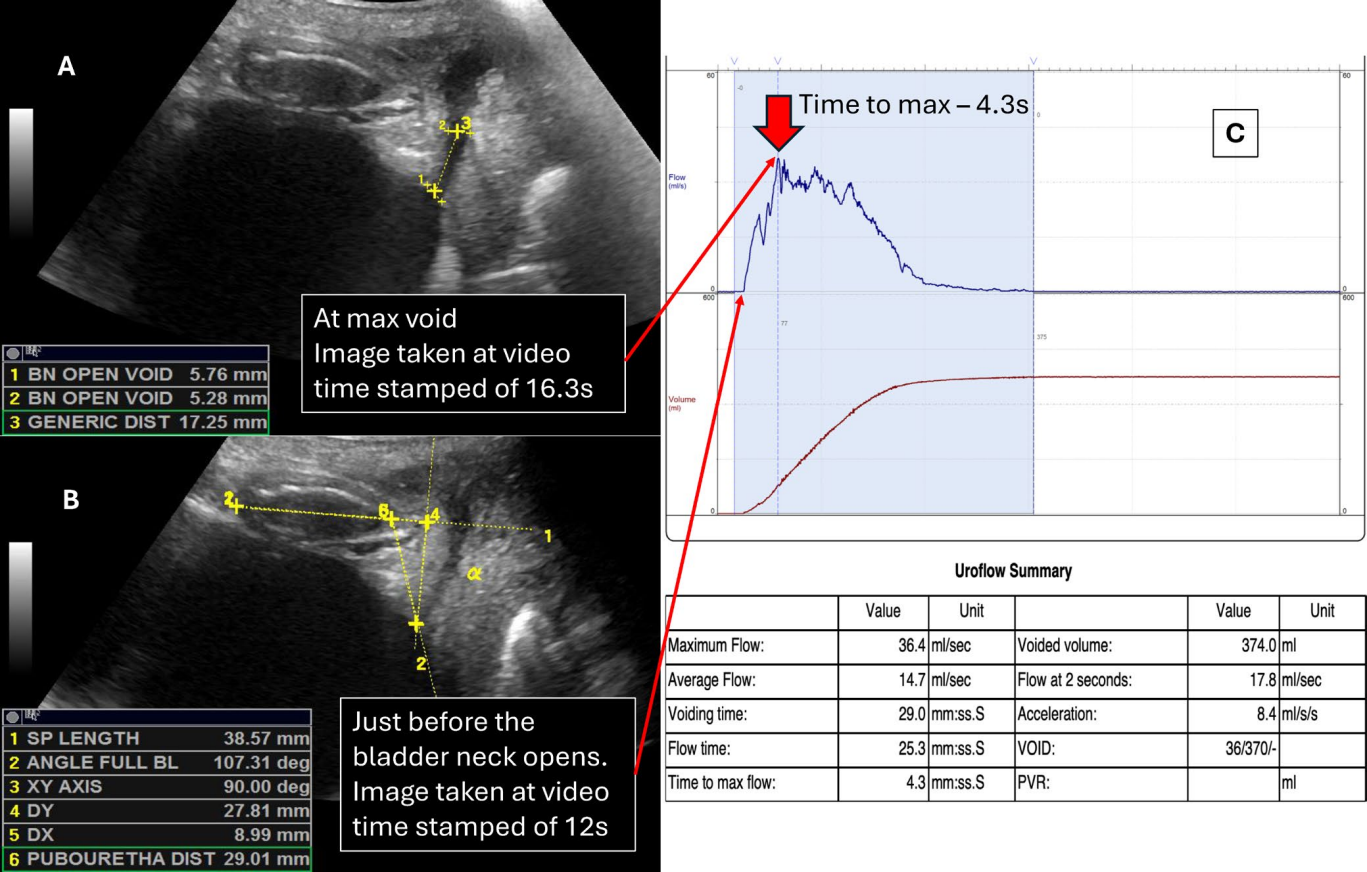
(A) Ultrasound image at timestamp 16.3 s with bladder neck and urethra opened at maximum void. Measures for bladder neck diameter (1) and urethral diameter (2) are shown. (B) Ultrasound image at timestamp 12 s where the bladder is full and just before the onset of bladder neck opening. (C) Uroflowmetry graph and parameters. The time of max flow is indicated by the red arrow.

The repeatability of new methods must be evaluated. Most measures showed good to excellent intra‐ and inter‐tester repeatability, except for BND and UD at the widest urethral diameter. Poor repeatability was attributed to variation in image selection and reference point. Initially, the frame with the widest urethral lumen was chosen subjectively. After a procedural change, repeatability improved by focusing on maximum flow from uroflowmetry. This illustrates the benefit of combining VS and uroflowmetry. The revised procedure for BND and UD measurements involved:

1 Image selection
  - The sonographer begins the VS recording once landmarks are identified and waits for the participant to void. The start of flow is marked when the bladder neck opens (Figure 2B). Note the timestamp on the VS video (i.e., 12 s).
  - The maximum flow is shown in Figure 2C, with the red arrow indicating 4.3 s.
  - Identify the duration between the onset and maximum void. From the VS, find the bladder neck opening (void onset, Panel B), then advance to the max flow timestamp of 16.3 s (Figure 2A) where BND and UD are measured.
2 Magnification and tool selection: The region of interest is enlarged, and the smallest caliper tool is used for measurement.
3 BND measurement: Calipers are placed on the inner walls of the ABNJ and PBNJ.
4 UD measurement: The measure is made 10 mm from the BNO midpoint, along a line parallel to the urethra (i.e., approximate location of the urethral striated muscle [23]). UD is measured perpendicular to the urethra between inner walls (lumen).

The SP joint space, used as the fixed landmark, is crucial for accurately assessing the BN and urethra during micturition. Although previous studies emphasize the importance of the sacrococcygeal to inferior pubis (SCIPP) line in a lateral pelvis position [24, 25, 26, 27], the joint space reported as 4.9 ± 1.9 mm [28] can be challenging to visualize in menopausal women and those with pathology. In our study, the repeatability of measuring the fixed landmark was fair.

Our study's ICC repeatability for Dx and Dy aligns with previous studies. A test–retest study of 17 healthy women showed good ICC (0.73) for Dx and very good ICC (0.8) for Dy. A study of male participants showed slightly better repeatability [29].

Several sources of variation exist between repeated measures, depending on the contexts and comparisons made. Common factors contributing to measurement errors include instrumentation, environmental conditions, procedural differences, and human factors [30]. As expected, our repeatability was best for measures by the same examiner or from the same image.

### 4.4 Methodological Considerations

This study has several potential limitations. First, the participants were pre‐menopausal, low BMI, and asymptomatic women, which may have improved measurement repeatability. Second, two women could not void, possibly due to discomfort or inadequate preparation, suggesting that this assessment may not suit all women. Third, the sample size limits precision, and larger studies would provide more accurate repeatability data. Fourth, protocol adjustments were made to improve the repeatability of the BND and UD measurements. Fifth, sonographer B had no prior VS experience and required training, potentially underpinning technique variations. Sixth, some uroflowmetry data were lost to urine spillage. Finally, manual matching of uroflowmetry and ultrasound parameters was necessary; using an integration software would improve accuracy.

## 5 Conclusion

VS, with the transducer placed in contact with the perineum towards the anterior perineum, provides repeatable voiding measures. Combined with uroflowmetry, VS shows promise for non‐invasive lower urinary tract assessment. Further research in symptomatic patients is needed to assess its clinical use, particularly when video fluoroscopy is unavailable or urodynamics fail to provide a diagnosis.

## Author Contributions

**Bernadette Dellar, Ryan Stafford, Eric Chung, and Paul W. Hodges:** conceptualization, methodology. **Bernadette Dellar, Anna Page, Handoo Rhee, Roxanna Turner, and Paul W. Hodges:** data curation. **Eric Chung and Paul W. Hodges:** supervision: **Bernadette Dellar:** writing – original draft preparation, investigation, software, funding acquisition, project administration. **Bernadette Dellar, Ryan Stafford, Eric Chung, Roxanna Turner, Handoo Rhee, Anna Page, Margret Sherburn, Gabriel Schaer, and Paul W. Hodges:** reviewing and editing. **Bernadette Dellar and Paul W. Hodges:** visualization, formal analysis. **Bernadette Dellar and Paul W. Hodges:** validation, final manuscript writing. All the authors commented on the manuscript.

## Ethics Statement

The study protocol was approved by the University of Queensland Human Research Ethics Committee. Decision no. 2021/HE000137 Date: December 14, 2021. All analysis performed involving human participants was in accordance with the 1964 Helsinki Declaration.

## Conflicts of Interest

The authors declare no conflicts of interest.

## Supporting information


**Data S1.** Classification of assessor's interpretation of flow pattern of Void 2 (no Tx on perineum) and Void 1 (with imaging Tx on perineum).
**Data S2**. Classification of assessor's interpretation of comparison of (same subject) flow pattern between Voids 1 and 3 (different days) and Voids 3 and 4 (same day).
**Data S3**. Results of Bland Altman limits of agreement and systematic bias results for ultrasound parameters (*n* = 32).
**Data S4**. Intra‐tester repeatability of sonographic measures of micturition between Void 1 and Void 3.
**Data S5**. Inter test repeatability of sonographic measures of micturition on the same images (Void 4) (*n* = 32).

## Data Availability

The data that support the findings of this study are available on request from the corresponding author. The data are not publicly available due to privacy or ethical restrictions.
