# Supplementary material for: A Repeatability Study of Measurement of Micturition With Voiding Sonography and Uroflowmetry of Asymptomatic Women
Source: Low Urin Tract Symptoms. 2025 Jul 8;17(4):e70020. doi: 10.1111/luts.70020 (PMC12238807; doi:10.1111/luts.70020)
Supplement: Supplementary file 1 — Data S1. Classification of assessor's interpretation of flow pattern of Void 2 (no Tx on perineum) and Void 1 (with imaging Tx on perineum). Data S2. Classification of assessor's interpretation of comparison of (same subject) flow pattern between Voids 1 and 3 (different days) and Voids 3 and 4 (same day). Data S3. Results of Bland Altman limits of agreement and systematic bias results for ultrasound parameters (n = 32). Data S4. Intra‐tester repeatability of sonographic measures of micturition between Void 1 and Void 3. Data S5. Inter test repeatability of sonographic measures of micturition on the same images (Void 4) (n = 32). [file LUTS-17-e70020-s001.docx]

**Supplementary Data 1** Classification of assessor’s interpretation of flow pattern of Void 2 (no Tx on perineum) and Void 1 (with imaging Tx on perineum)

| **ID** | **BD** | **HR** | **AP** | **BD** | **HR** | **AP** | **Between assessor’s classification** | **Between tasks** |
| --- | --- | --- | --- | --- | --- | --- | --- | --- |
|  | **Void 2**  **(no imaging)** | | | **Void 1** | | |  |  |
| **1** | 1 | 1 | 4 | 1 | 1 | 4 | Minor | Agree |
| **2** | 1 | 1 | 1 | 5 | 5 | 5 | Agree | Major |
| **3** | 1 | 1 | 3 | 1 | 1 | 1 | Minor | Agree |
| **4** | Missing data | | | | | | | |
| **5** | 2 | 2 | 2 | 2 | 2 | 2 | Agree | Agree |
| **6** |  |  |  |  |  |  |  |  |
| **7** | Missing data | | | | | | | |
| **8** | 1 | 1 | 1 | 1 | 1 | 1 | Agree | Agree |
| **9** | 2 | 2 | 1 | 2 | 1 | 1 | Minor | Minor |
| **10** | 5 | 5 | 5 | 5 | 5 | 5 | Agree | Agree |
| **11** | 4 | 3 | 4 | 4 | 3 | 4 | Minor | Agree |
| **12** | 1 | 1 | 1 | 1 | 1 | 1 | Agree | Agree |
| **13** | 1 | 1 | 1 | 1 | 1 | 1 | Agree | Agree |
| **14** | 1 | 1 | 1 | 1 | 1 | 1 | Agree | Agree |
| **15** | 2 | 2 | 2 | 2 | 2 | 2 | Agree | Agree |
| **16** | 1 | 1 | 1 | 1 | 5 | 3 | Major | Major |
| **17** | 3 | 5 | 3 | 3 | 3 | 5 | Minor | Minor |
| **18** | 1 | 1 | 3 | 1 | 5 | 1 | Minor | Minor |
| **19** | 1 | 1 | 1 | 1 | 1 | 1 | Agree | Agree |
| **20** | 1 | 2 | 1 | 4 | 4 | 3 | Minor | Major |
| **21** | 1 | 1 | 1 | 5 | 3 | 5 | Minor | Major |
| **22** | 5 | 5 | 5 | 5 | 5 | 5 | Agree | Agree |
| **23** | 5 | 5 | 5 | 5 | 5 | 5 | Agree | Agree |
| **24** | 1 | 2 | 2 | 1 | 1 | 2 | Minor | Minor |
| **25** | 1 | 2 | 1 | 1 | 1 | 1 | Minor | Minor |
| **26** | 1 | 2 | 1 | 1 | 3 | 3 | Minor | Minor |
| **27** | 2 | 2 | 3 | 2 | 2 | 1 | Minor | Minor |
| **28** | 5 | 3 | 5 | 5 | 5 | 4 | Minor | Minor |
| **29** | 3 | 3 | 3 | 3 | 4 | 3 | Minor | Minor |
| **30** | 2 | 2 | 1 | 2 | 1 | 3 | Major | Minor |
| **31** |  |  |  |  |  |  |  |  |
| **32** | 1 | 1 | 1 | 1 | 2 | 3 | Major | Minor |
| **33** | 1 | 3 | 3 | 1 | 1 | 1 | Minor | Major |
| **34** | 1 | 1 | 3 | 4 | 4 | 3 | Major | Major |
| **35** |  |  |  |  |  |  |  |  |

Uroflow Shapes: 1-Bell, 2-Tower, 3-Undulated/staccato, 4-Interrupted, 5-Plateau; Minor difference - flow patterns changed from 3 to 4;1 to 2 or if the pattern difference identified by only 1 assessor; Major difference - change from 1/2, to 3/4/5, and differences categorised by 2 assessors. BD – Bernadette Dellar, HR – Handoo Rhee, AP – Anna Page.

**Supplementary Data 2** Classification of assessor’s interpretation of comparison of (same subject) flow pattern between voids 1 and 3 (different days) and voids 3 and 4 (same day).

|  | ***BD*** | ***HR*** | ***AP*** | ***BD*** | ***HR*** | ***AP*** | ***BD*** | ***HR*** | ***AP*** | ***Comparison between*** | |
| --- | --- | --- | --- | --- | --- | --- | --- | --- | --- | --- | --- |
| **ID** | **Void 1** | | | **Void 3** | | | **Void 4** | | | **Void 1 & 3** | **Void 3 & 4** |
| 1 | 1 | 1 | 4 | 5 | 1 | 1 | 5 | 1 | 1 | Major | Major |
| 2 | 5 | 5 | 5 | 5 | 5 | 5 | 5 | 3 | 5 | Agree | Minor |
| 3 | 1 | 1 | 3 | 1 | 2 | 1 | 1 | 1 | 1 | Major | Minor |
| 4 | Missing data | | | 4 | 3 | 3 | 4 | 4 | 4 | - | Minor |
| 5 | 2 | 2 | 2 | 3 | 3 | 3 | 2 | 2 | 2 | Major | Major |
| 6 |  |  |  |  |  |  |  |  |  |  |  |
| 7 | Missing data | | | 3 | 3 | 3 | 4 | 4 | 4 | - | Minor |
| 8 | 1 | 1 | 1 | 1 | 1 | 1 | 1 | 1 | 1 | Agree | Agree |
| 9 | 2 | 1 | 1 | 2 | 2 | 1 | 2 | 1 | 1 | Minor | Minor |
| 10 | 5 | 5 | 5 | 5 | 4 | 5 | 5 | 5 | 5 | Minor | Minor |
| 11 | 4 | 3 | 4 | 4 | 3 | 3 | 4 | 4 | 4 | Minor | Minor |
| 12 | 1 | 1 | 1 | 1 | 1 | 1 | 1 | 1 | 1 | Agree | Agree |
| 13 | 5 | 1 | 1 | 3 | 5 | 3 | 4 | 1 | 3 | Major | Major |
| 14 | 1 | 1 | 1 | 1 | 1 | 1 | 1 | 1 | 3 | Agree | Agree |
| 15 | 2 | 2 | 2 | 2 | 2 | 2 | 2 | 2 | 2 | Agree | Agree |
| 16 | 1 | 5 | 3 | 5 | 5 | 5 | 5 | 5 | 3 | Major | Minor |
| 17 | 3 | 3 | 5 | 3 | 4 | 5 | 3 | 3 | 5 | Minor | Major |
| 18 | 1 | 5 | 1 | 5 | 5 | 5 | 5 | 5 | 5 | Major | Agree |
| 19 | 1 | 1 | 1 | 1 | 1 | 3 | 1 | 1 | 1 | Minor | Minor |
| 20 | 4 | 4 | 3 | 4 | 4 | 4 | 3 | 3 | 3 | Minor | Minor |
| 21 | 3 | 3 | 5 | 3 | 5 | 3 | 3 | 3 | 3 | Minor | Minor |
| 22 | 5 | 5 | 5 | 5 | 5 | 5 | 5 | 5 | 5 | Agree | Agree |
| 23 | 5 | 5 | 5 | 5 | 3 | 5 | 5 | 5 | 1 | Minor | Major |
| 24 | 1 | 1 | 2 | 3 | 1 | 3 | 1 | 1 | 3 | Major | Major |
| 25 | 1 | 1 | 1 | 1 | 2 | 1 | 1 | 2 | 1 | Minor | Minor |
| 26 | 1 | 3 | 3 | 3 | 1 | 1 | 1 | 1 | 1 | Major | Minor |
| 27 | 2 | 2 | 1 | 2 | 2 | 3 | 2 | 2 | 2 | Minor | Minor |
| 28 | 5 | 5 | 4 | 5 | 5 | 4 | 4 | 5 | 5 | Agree | Minor |
| 29 | 3 | 4 | 3 | 3 | 3 | 3 | 3 | 3 | 3 | Minor | Agree |
| 30 | 2 | 1 | 3 | 3 | 1 | 3 | 5 | 1 | 3 | Major | Major |
| 31 |  |  |  |  |  |  |  |  |  |  |  |
| 32 | 1 | 2 | 3 | 1 | 1 | 1 | 1 | 1 | 1 | Minor | Agree |
| 33 | 1 | 1 | 1 | 1 | 3 | 3 | 1 | 3 | 3 | Minor | Agree |
| 34 | 4 | 4 | 3 | 1 | 1 | 1 | 5 | 1 | 1 | Major | Minor |
| 35 |  |  |  |  |  |  |  |  |  |  |  |

Uroflow Shapes: 1-Bell, 2-Tower, 3-Undulated/staccato, 4-Interrupted, 5-Plateau; Minor difference - flow patterns changed from 3 to 4;1 to 2 or if the pattern difference identified by only 1 assessor; Major difference - change from 1/2, to 3/4/5, and differences categorised by 2 assessors. BD – Bernadette Dellar, HR – Handoo Rhee, AP – Anna Page.

**Supplementary data 3** Results of Bland Altman limits of agreement and systematic bias results for ultrasound parameters (n=32)

|  | Parameter | | Void 1 vs. Void 3 (95% CI) | | Bias | | Void 3 vs. Void 4 (95% CI) | | Bias | | BD vs RT (95% CI) | | Bias | |
| --- | --- | --- | --- | --- | --- | --- | --- | --- | --- | --- | --- | --- | --- | --- |
| At full Bladder | SP (mm) | -6.8 to 5.9 | | -0.5 | | -8.9 to 6 | | -1.5 | | -7.0 to 3.7 | | -1.6 | |  |
|  | PUA (degrees) | -25.2 to 29.9 | | 2.3 | | -30 to 19.3 | | -5.3 | | -17.9 to 10.8 | | -3.6 | |  |
|  | S-BN (mm) | -5.9 to 4.9 | | -0.5 | | -4.3 to 5.3 | | 0.5 | | -3.5 to 4.9 | | 0.7 | |  |
|  | Dx (mm) | -10.4 to 13.1 | | 1.4 | | -10.9 to 8.6 | | -1.2 | | -7.0 to 6.2 | | -0.4 | |  |
|  | Dy (mm) | -10.6 to 7.8 | | -1.4 | | -5.2 to 7.7 | | 1.3 | | -5.7 to 7.6 | | 0.9 | |  |
| At max urethral diameter | SP (mm) | -6.8 to 6 | | -0.4 | | -8.1 to 5.5 | | -1.3 | | -5.7 to 3.3 | | -1.2 | |  |
|  | PUA (degrees) | -23.9 to 22.8 | | -0.5 | | -20.4 to 20.6 | | 0.1 | | -16.2 to 13.6 | | -1.3 | |  |
|  | S-BN (mm) | -6.7 to 7.1 | | 0.2 | | -5.4 to 6.9 | | 0.8 | | -5.8 to 7.5 | | 0.9 | |  |
|  | Dx (mm) | -8.7 to 9.1 | | 0.2 | | -8.9 to 9 | | 0.1 | | -6.5 to 5.6 | | -0.4 | |  |
|  | Dy (mm) | -8.7 to 9.1 | | 0.2 | | -5.4 to 6.7 | | 0.7 | | -4.4 to 6.2 | | 0.9 | |  |
| At max flow | BND (mm) | -8.0 to 6.9 | | -0.6 | | -6.4 to 5.0 | | -0.7 | | -3.2 to 4.3 | | 0.6 | |  |
|  | UD (mm) | -4.3 to 3.7 | | -0.3 | | -4.4 to 4 | | -0.2 | | -2.3 to 3 | | 0.4 | |  |
| At end void | SP (mm) | -10.2 to 6.8 | | -1.7 | | -8.0 to 9.1 | | 0.6 | | -7.0 to 6.5 | | -0.3 | |  |
|  | PUA (degrees) | -21.0 to 21.5 | | 0.27 | | -31.9 to 26.7 | | -2.6 | | -17.3 to 12.4 | | -2.5 | |  |
|  | S-BN (mm) | -5.5 to 7.2 | | 0.9 | | -5.6 to 6.2 | | 0.3 | | -3.4 to 5.2 | | 0.9 | |  |
|  | Dx (mm) | -9.1 to 8.9 | | -0.1 | | -11.2 to 10.3 | | -0.5 | | -6.5 to 5.8 | | -0.3 | |  |
|  | Dy (mm) | -15.1 to 17 | | 1 | | -8.0 to 10.6 | | 1.3 | | -4.5 to 7 | | 1.3 | |  |

BD – Bernadette Dellar; RT – Roxanne Turner

Void 1 (performed on day1 by BD), Void 3 (performed on day2 by BD) and Void 4 (performed on day2 by RT)

**Supplementary data 4** Intra-tester repeatability of sonographic measures of micturition between Void 1 and Void 3

|  | Parameter | Void 1 Average (Range) median (SD) | Void 3 Average (Range) median (SD) | ICC |
| --- | --- | --- | --- | --- |
| At full bladder | SP (mm) | 38.2 (30.6 – 46.8) 37.6 (4.1) | 38.7 (30 – 46.3) 38.3 (3.4) | 0.63 |
|  | PUA (degrees) | 120 (79.3 – 154.7) 118.7 (17.8) | 118 (87.4 – 163.5) 117.4 (17.5) | 0.68 |
|  | S-BN (mm) | 26 (16.6 – 32.1) 26 (3.3) | 26.5 (21.5 – 36) 26.1 (3.7) | 0.68 |
|  | Dx (mm) | 13.5 (-4.5 – 27.6) 14.1 (6.8) | 12.2 (-1.2 – 30.2) 11.6 (7) | 0.62 |
|  | Dy (mm) | 20.8 (9.2 – 27.8) 21.8 (5.2) | 22.1 (8.7 – 30.9) 22.7 (5.1) | 0.57 |
| At widest urethral diameter | SP (mm) | 37.9 (31.7 – 45.1) 38.4 (3.5) | 38.3 (32 – 45) 37.8 (3.2) | 0.53 |
|  | PUA (degrees) | 121.4 (68.1 – 156.5) 121.2 (17.9) | 121.9 (69.7 – 172.4) 69.7 (19.4) | 0.80 |
|  | S-BN (mm) | 24.7 (9.9 – 31.6) 25.2 (4.2) | 24.5 (18.2 – 31.6) 24.1 (3.6) | 0.61 |
|  | Dx (mm) | 12.7 (-8.6 – 24.7) 13.7 (6.7) | 12.5 (-8.3 – 29.5) 13.3 (6.9) | 0.78 |
|  | Dy (mm) | 19.7 (3.9 – 27.6) 21.1 (5.7) | 19.5 (4 – 28.4) 20.8 (5.4) | 0.67 |
| At max flow | BND (mm) | 7.4 (1.4 – 17) 7.2 (3.6) | 7.8 (2.8 – 13.3) 7.5 (2.8) | 0.34 |
|  | UD (mm) | 4.9 (2.2 – 8) 4.7 (1.6) | 5.1 (1.0 – 9.3) 5.2 (2.3) | 0.39 |
| At end void | SP (mm) | 38.1 (31.1 – 45) 38.3 (3.8) | 39.8 (32 – 46.2) 40.4 (3.9) | 0.35 |
|  | PUA (degrees) | 125.3 (81.2 – 155.3) 125.1 (18.4) | 125.1 (81.8 – 156.5) 126.3 (17.3) | 0.82 |
|  | S-BN (mm) | 27.1 (17.4 – 34.7) 27.4 (4) | 26.3 (19.2 -33) 26.3 (3.3) | 0.60 |
|  | Dx (mm) | 14.6 (-5 – 26.3) 16.4 (6.9) | 14.7 (-3.2 – 29.4) 15.7 (7) | 0.79 |
|  | Dy (mm) | 21.1 (11.2 – 34.4) 29.9 (6.4) | 20.8 (10.9 – 32) 21.2 (5.2) | 0.77 |

Void 1 (performed on day1 by BD), Void 3 (performed on day2 by BD) and Void 4 (performed on day2 by RT) Poor - <0.20, fair - 0.21-0.40, moderate - >0.40-0.60, good - 0.60-0.80 and very good or excellent - 0.81-1.00

**Supplementary data 5** Inter test repeatability of sonographic measures of micturition on the same images (Void 4) (n=32)

|  | Parameter | BD Average (Range) median (SD) | RT Average (Range) median (SD) | ICC |
| --- | --- | --- | --- | --- |
| At full bladder | SP (mm) | 38.5 (33 – 45.7) 38.6 (2.8) | 40.1 (35.1 – 45) 39.9 (2.6) | 0.42 |
|  | PUA (degrees) | 119.7 (73.3 – 155.1) 118.8 (19.4) | 123.3 (79.6 – 160.6) 123.4 (17.6) | 0.91 |
|  | S-BN (mm) | 26.6 (20.8 – 33.7) 25.8 (3.6) | 26 (19.1 – 33.5) 25.9 (3.6) | 0.82 |
|  | Dx (mm) | 12.9 (-6.8 – 28) 14.3 (7.4) | 13.3 (-5.1 – 28.7) 13.5 (7) | 0.89 |
|  | Dy (mm) | 21.7 (12 – 30) 22.6 (5.2) | 20.9 (10.5 – 28.4) 21.8 (4.8) | 0.76 |
| At widest urethral diameter | SP (mm) | 38.4 (31 – 44.3) 38.8 (3.4) | 39.5 (32.6 – 44.6) 39.8 (2.7) | 0.68 |
|  | PUA (degrees) | 120.6 (64 – 170.2) 119.1 (20.5) | 121.9 (69.4 – 163.1) 121.1 (19.3) | 0.93 |
|  | S-BN (mm) | 38.4 (31 – 44.3) 38.8 (3.4) | 23.8 (12.1 – 31) 23.6 (4) | 0.63 |
|  | Dx (mm) | 12 (-9.4 – 25.9) 11.6 (7) | 12.5 (-8.2 – 29.5) 12.4 (7.5) | 0.91 |
|  | Dy (mm) | 19.7 (4.6 – 29) 20.7 (5.9) | 18.9 (9.3 – 26.7) 19.6 (4.6) | 0.86 |
| At max flow | BND (mm) | 8.2 (3 – 13.3) 7.5 (2.8) | 8.8 (4.2 – 15.1) 8.5 (2.1) | 0.70 |
|  | UD (mm) | 5.1 (1.0 – 9.3) 5.2 (2.3) | 5.4 (4.2 – 9.1) 5.1 (1.5) | 0.63 |
| At end void | SP (mm) | 39 (31.5 – 45.9) 38.9 (3.6) | 39.3 (31.8 – 45.7) 39.2 (2.8) | 0.44 |
|  | PUA (degrees) | 125.2 (80.4 – 161.5) 128.7 (18.3) | 127.7 (85.3 – 155.7) 128.6 (18.4) | 0.91 |
|  | S-BN (mm) | 26.9 (19.5 – 34.1) 26.7 (3.8) | 26 (18.6 – 33.9) 25.9 (4.1) | 0.83 |
|  | Dx (mm) | 14.9 (-3.7 – 26.9) 15.9 (7.1) | 15.2 (-1.5 -27.7) 16.2 (6.6) | 0.90 |
|  | Dy (mm) | 20.7 (10.2 – 31.1) 21 (5.4) | 19.5 (9.6 – 32) 19.3 (6.1) | 0.85 |

Void 1 (performed on day1 by BD), Void 3 (performed on day2 by BD) and Void 4 (performed on day2 by RT) Poor - <0.20, fair - 0.21-0.40, moderate - >0.40-0.60, good - 0.60-0.80 and very good or excellent - 0.81-1.00
